# Supplementary material for: Direct Single-Cell Analysis of Human Polar Bodies and Cleavage-Stage Embryos Reveals No Evidence of the Telomere Theory of Reproductive Ageing in Relation to Aneuploidy Generation
Source: Cells. 2019 Feb 16;8(2):163. doi: 10.3390/cells8020163 (PMC6406255; doi:10.3390/cells8020163)
Supplement: Supplementary file 1 [file cells-08-00163-s001.pdf]

Patient ID	Age	Total number of polar bodies assessed	Number of an-euploid polar bodies assessed	Chromosomes in-volved in aneuploid polar body number 1	Chromosomes in-volved in aneuploid polar body number 2
1	34	3	1	+4	n/a
2	38	4	2	-16	+4, -15
4	33	4	2	-17	-16
7	41	4	2	+10	+16
35	40	4	2	+22	complex
41	33	4	2	complex	+20
45	31	4	1	+22,+19,+21	n/a
46	42	4	2	-11	+16
50	33	4	0	n/a	n/a
52	26	4	0	n/a	n/a
59	41	4	2	complex	+16,-9,-21
60	37	4	2	+22	+12,+22
66	33	3	2	-4	+16
70	33	2	1	+18,+X	n/a
72	31	4	1	-22	n/a
73	43	4	2	complex	+1,+7,+10
74	47	3	2	+15,+18	+11,+20,+22
75	36	4	2	-2,-8	-22
76	33	2	0	n/a	n/a
80	32	3	1	+11	n/a
82	37	4	2	+15,+21	+21
84	37	4	2	-12,-20	complex
91	32	4	0	n/a	n/a
103	31	3	1	+9	n/a
115	46	3	2	complex	complex

Table S1: Anonymised patient details and results of aneuploidy screening in first polar bodies following assessment using the 24 Sure preimplantation genetic screening kit. 'complex' refers to aneuploid results involving four or more chromosomes, 'n/a' is not applicable (i.e. no aneuploid polar body available).

Patient ID	Age	Total number of blastomeres assessed	Number of aneuploid blastomeres assessed	Chromosomes involved in aneuploid blastomere number 1	Chromosomes involved in aneuploid blastomere number 2
19	34	4	2	-18, -21	-22
20	43	4	2	XO	dup(13)(q21.31-qter),+16
21	37	4	2	-13,+14	-15
22	41	4	2	-16	complex
24	42	4	2	complex	complex
27	34	4	2	complex	del(11)(q23.3-qter)
33	36	4	2	45;-9	47;+16
34	33	4	2	del(8)(q22.1-qter),-22	-8, del(15)(q25.1-qter)
122	38	4	2	del(1)(p21.1-pter),+22	del(2)(q33.3-qter),-15
123	40	4	2	complex	+19
127	39	4	2	-17,+18	complex
129	42	4	2	+10,+19	complex
130	34	4	2	+13	complex
137	32	4	2	del(6)(p12.1-pter), del(13)(q31.3-qter), del(16)(q11.2-qter)	complex
143	41	4	2	complex	44;-4,-21
144	33	4	2	+14,+22	del(1)(p32.3-pter),-10
163	33	4	2	+5,+21,+22	complex
171	27	4	2	dup(1)(p12-qter),+21	complex
172	34	4	2	complex	-4
188	33	4	2	-5	complex
224	44	2	1	complex	n/a
225	30	4	2	complex	complex

Table S2: Patient details and results of aneuploidy screening in blastomeres biopsied from cleavage stage embryos following assessment using the 24 Sure preimplantation genetic screening kit. ‘complex’ refers to aneuploid results involving four or more chromosomes, ‘n/a’ is not applicable (i.e. no embryo available), ‘del’ refers to a deletion with the chromosome involved identified in the first set of brackets and the portion deleted identified in the second brackets, ‘dup’ refers to a duplication with the chromosome involved identified in the first set of brackets and the portion duplicated identified in the second brackets.
